# Body Composition, Physical Activity, and Convenience Food Consumption among Asian American Youth: 2011–2018 NHANES

**DOI:** 10.3390/ijerph17176187

**Published:** 2020-08-26

**Authors:** Soyang Kwon, Meme Wang-Schweig, Namratha R. Kandula

**Affiliations:** 1 Ann & Robert H. Lurie Children’s Hospital of Chicago, Chicago, IL 60611, USA; 2 Division of Community Health Sciences, University of Illinois Chicago School of Public Health, Chicago, IL 60607, USA; lwang33@uic.edu; 3 Department of Medicine and Preventive Medicine, Northwestern University, Chicago, IL 60611, USA; n-kandula@northwestern.edu

**Keywords:** acculturation, obesity, muscle strength, children, adolescents

## Abstract

The primary purpose of this study was to describe obesity, body composition, convenience food consumption, physical activity, and muscle strength among Asian American youth compared to other racial/ethnic groups. The secondary purpose was to examine whether obesity, body composition, convenience food consumption, physical activity, and muscle strength differed by acculturation levels among Asian American youth. A secondary analysis was conducted using data from 12,763 children aged 2 to 17 years that participated in the 2011–2018 US National Health and Nutrition Examination Survey (NHANES). In the NHANES interview, acculturation, dietary behavior, and physical activity questionnaires were administered. The acculturation level was indicated by the language spoken at home. In the NHANES examination, anthropometry, dual-energy X-ray absorptiometry (DXA), and muscle strength assessments were conducted. Compared to non-Hispanic White American boys, Asian American boys had similar levels of obesity, central obesity, and fat mass. Among the five racial/ethnic groups examined, lean body mass, muscle mass, convenience food consumption, and daily physical activity were the lowest in the Asian group. More acculturated Asian American boys, but not girls, were more likely to be obese (OR = 3.28 (1.63, 6.60)). More acculturated Asian American youth more frequently consumed convenience food (1.4 more meals/month (1.2, 1.6)). This study highlights the obesity problem among Asian American boys, which worsens with acculturation to America. The study results also suggest that although Asian American youth consume less convenience food overall than non-Hispanic White American youth, increasing acculturation may negatively influence food choices.

## 1. Introduction

Asian Americans account for approximately 6% of the US population [1] and are the fastest-growing major racial/ethnic group in the US, growing 72% between 2000 and 2015 [2]. The Asian group included all persons having origins in the Far East, Southeast Asia, or the Indian subcontinent, including China, Japan, Korea, Malaysia, the Philippine Islands, Vietnam, Thailand, Cambodia, India, and Pakistan [3,4]. Although Asian Americans disproportionally suffer from certain types of disease [5], they have similar life expectancies (79.9 years) to non-Hispanic White Americans (79.8 years) [1] or even better health measures, such as a lower childhood obesity prevalence [6] and a healthier diet [7]. Traditional Asian diets are rich in fruits and vegetables, legumes, and fish, and are low in fat [8,9], which are a favorable dietary pattern for obesity prevention [9,10,11]. However, research suggests that acculturation to the US plays a significant role in the increased risk of obesity and other chronic diseases among Asian American adults [12,13]. Acculturation is the process by which immigrants adapt to their new host country [14]. Although nationally representative data are lacking, a limited number of local/regional studies have shown that Asian American youth, across different Asian ethnic subgroups, increasingly consume diets higher in saturated fat, trans fat, sweets, and soda as they become acculturated [15,16]. Unger and colleagues [17] also demonstrated that acculturation is associated with a higher frequency of fast food consumption among Asian American adolescents, which is linked to higher energy intake and an increased risk of obesity [18,19].

Regarding physical activity as a critical behavior for preventing obesity, the relationship between acculturation and physical activity among Asian American youth is rarely studied and the results are less clear: while Allen and colleagues [20] showed that engagement in physical activity increased with increasing generations in the US, Unger and colleagues [17] showed that acculturation was associated with less frequent participation in physical activity. Physical activity guidelines for American youth aged 6 to 17 years recommend 60 minutes of moderate- to vigorous-intensity physical activity daily, part of which should include 3 days of muscle-strengthening physical activity and 3 days of bone-strengthening physical activity a week [21]. The extent to which Asian American youth meet these recommendations is currently unknown.

In the midst of increasing trends of childhood obesity in the US [22], as well as in Asian nations [23,24], it is important to provide epidemiological data on the prevalence of obesity and obesity-related behaviors (i.e., unhealthy diet and physical inactivity) among Asian American youth to facilitate the prevention and control of childhood obesity. However, such a national evaluation was significantly limited by the lack of nationally representative data. Recognizing the growth of the Asian American population, the US National Health and Examination Survey (NHANES) began to oversample Asian Americans and separately categorize the “Asian” ethnicity, rather than categorizing Asian individuals into the “other” race/ethnicity group in 2011. Thus, the NHANES now provides nationally representative data that can be used to evaluate health conditions and health-related factors in the Asian American population. Utilizing the 2011–2018 NHANES data, this study aimed to describe the obesity, body composition, convenience food consumption, physical activity, and muscle strength among Asian American youth relative to their racial/ethnic counterparts. The secondary purpose was to examine whether obesity, body composition, convenience food consumption, physical activity, and muscle strength differed by acculturation levels among Asian American youth.

## 2. Methods

### 2.1. Study Sample

A secondary analysis was conducted using the 2011–2012, 2013–2014, 2015–2016, and 2017–2018 US NHANES datasets. The NHANES target population is the noninstitutionalized civilian resident population of the US. In each cycle, the NHANES examines a representative sample of the US population using a complex staged sampling method. Beginning in 2011, NHANES oversampled non-Hispanic Asians, in addition to the ongoing oversampling of Hispanics, non-Hispanic Blacks, older adults, and low-income Whites/others. The sample of this study included 12,763 youth aged 2 to 17 years who participated in the 2011–2018 NHANES (*n* = 3271 in years 2011–2012, *n* = 3395 in years 2013–2014, *n* = 3290 in years 2015–2016, *n* = 2807 in years 2017–2018). Of those included, 1301 were Asian, 3397 were non-Hispanic White, 3901 were Hispanic, 3247 were non-Hispanic Black, and 917 were of other races.

### 2.2. Measurements

The NHANES is largely comprised of two assessments: an interview and an examination. An in-person interview was conducted in each participant’s home by trained interviewers using the Computer-Assisted Personal Interview (CAPI) system. For child participants aged 15 years or younger or a participant who could not answer the questions themselves, a proxy provided information for the participant. The respondents selected the spoken language of the interview (English or Spanish) or requested that an interpreter be used. To facilitate the oversampling of the Asian population, survey materials (e.g., hand cards, glossaries of terms, consent forms, examination scripts, and informational brochures) were translated into Mandarin Chinese (both traditional and simplified), Korean, and Vietnamese. Anything the participant completed on his/her own, such as the automated computer-assisted self-interviewing interview, was translated and recorded into an Asian language [25]. The NHANES staff participated in cultural competency training to help them recognize and respect cultural differences. Local interpreters, as well as professional medical interpreter phone services, were available to assist with any language needs. During the interview, demographics, acculturation, dietary behavior, and physical activity questionnaires were administered. In the demographic questionnaire, participants self-identified their Hispanic origin, where applicable. They also selected one or more self-identified race among the following options: White, Black or African American, Asian, American Indian or Alaska Native, Native Hawaiian or Pacific Islander, or other. The Hispanic origin and race responses were used to categorize race/ethnicity into the following five categories: Hispanic, non-Hispanic White, non-Hispanic Black, non-Hispanic Asian, or other race, including multi-racial. Although the Asian origin (e.g., Chinese, Korean, Vietnamese) was reported, this study could not examine the Asian subgroups due to there being a small sample size for each subgroup [25].

Based on the family size and family income reported in the demographic questionnaire, a ratio of family income to poverty (poverty index ratio) was calculated. The poverty index ratio was categorized into low (<1.0), middle (1 to <3.0), and high (≥3.0). For example, for a family size of four, including two children under age 18 years, the poverty threshold in 2012 was $23,283. Therefore, the threshold of 3 for the poverty index ratio was equal to $69,849 for a family size of four in 2012. The acculturation questionnaire, which was administered in a subsample (≥12 years old in 2011 and ≥3 years old in 2012–2018), contained only one question item asking about the language spoken at home, with the following five response options: English language only, more English than non-English, both languages equally, more non-English than English, or non-English language only at home. To use the language spoken at home as an indicator of acculturation [26], the home language response was categorized into three groups: (1) English only (more acculturated), (2) more English or both languages equally (middle), and (3) more non-English or non-English only (less acculturated). We additionally explored the self-reported birthplace (US or non-US) as an acculturation indicator.

In the dietary behavior questionnaire, this study focused on convenience food consumption, namely, the number of frozen meals or frozen pizzas eaten in the past 30 days. In the physical activity questionnaire, participants were asked how many days in the past 7 days they were physically active for a total of at least 60 minutes per day. Based on the aerobic Physical Activity Guidelines for American youth aged 6 to 17 years, which recommends ≥60 minutes of moderate- to vigorous-intensity physical activity daily [21], the responses were dichotomized into 7 days (daily physical activity) and <7 days (no daily physical activity).

Physical examinations were conducted in the Mobile Examination Center, which consisted of a single mobile trailer. During the examination, standing height, weight, and waist circumference were measured by trained health technicians. Body mass index (BMI) was calculated as weight (kg) divided by height squared (m^2^). Sex- and age-specific BMI percentiles were calculated based on the 2000 US Centers for Disease Control and Prevention (CDC) growth charts [27]. The BMI percentiles were then dichotomized as ≥95th percentile (obesity) and <95th percentile. Based on the international sex- and age-specific waist circumference percentile reference [28], participants aged 6 years and older were categorized into ≥95 percentile (central obesity) and <95th percentile. Dual-energy X-ray absorptiometry (DXA) whole-body scans were administered to participants aged 8 years and older using Hologic Discovery model A densitometers (Hologic, Inc.; Bedford, MA, USA). Pregnant females were excluded from the DXA examination. The scan images were analyzed using APEX version 4.0 software (Hologic, Inc.; Bedford, MA, USA) with the NHANES body composition analysis (BCA) option, which added 5% of lean mass to the fat mass to correct the underestimation of fat mass by this particular densitometer [29]. From this analysis, total fat mass, total lean mass, and bone mass content were derived. The fat mass index (FMI) was calculated as total fat mass (kg) divided by height squared (m^2^). The lean body mass index (LBMI) was calculated as total lean mass excluding bone mass content (kg) divided by height squared (m^2^). Based on the sex- and age-specific FMI percentile references [30], participants were categorized into ≥75 percentile (elevated FMI) and <75th percentile [30]. Based on the sex- and age-specific LBMI percentile references [30], participants were categorized into ≤25th percentile (low LBMI) and >25th percentile. To assess muscle strength, grip tests were conducted using handgrip dynamometers among participants aged 6 years and older only in the 2011–2014 NHANES, but not in the 2015–2018 NHANES. Grip strength is a validated measure for evaluating general muscle strength in youth [31]. Participants were excluded from the grip test component if they were unable to hold the dynamometer with both hands (e.g., missing both arms, both hands, thumbs on both hands, or paralyzed in both hands) or if they had surgery on either hand or wrist in the last three months. The grip test was performed in the standing position unless the participant was physically limited. A trained examiner adjusted the grip size of the dynamometer to the participant’s hand size and asked the participant to squeeze the dynamometer for a practice trial to ensure that the grip size was properly adjusted. After the practices, the participant was asked to use one of the hands to squeeze the dynamometer as hard as possible. The participant was randomly assigned to start the test with his/her dominant or non-dominant hand. The test was then repeated with the other hand. Each hand was tested three times, alternating hands between trials with a 60-second rest between measurements on the same hand. The largest reading from each hand was summed to indicate the grip strength in kilograms.

### 2.3. Statistical Analysis

Unweighted sample sizes were calculated by race/ethnicity, age group, and sex. All analyses were conducted by accounting for the complex sample design, such as weighting and clustering. The sample weight was created in three steps: (1) the base weight that accounted for the unequal probability of selection, (2) adjustment for non-response, and (3) post-stratification adjustment to match estimates of the US civilian non-institutionalized population available from the Census Bureau. Missing data were treated using a listwise deletion method. Chi-square tests were conducted to compare the demographic characteristics of those who were excluded to those who were included. Descriptive analyses, including means and 95% confidence intervals (CIs), were conducted by race/ethnicity, age group (2 to 5, 6 to 11, and 12 to 17 years), and sex. To examine whether the acculturation level was associated with the binary outcomes, such as obesity, central obesity, elevated FMI, low LBMI, and daily physical activity in Asian American youth, multivariable logistic analyses were conducted and adjusted for key demographic factors, such as sex, age group, and family income level. For the continuous outcomes, such as convenience food consumption (meals/month) and muscle strength (kg), multivariable linear regression analyses were conducted. To examine the potential sex difference in the impact of acculturation, we conducted additional sex-specific regression analyses. When sex was found to be a modifier, sex-specific regression analysis results were presented. All analyses were performed using the SAS 9.4 survey procedure (Cary, NC, USA).

## 3. Results

Table 1 presents the unweighted sample sizes by race/ethnicity, age group, and sex. In the subsample of 7413 participants aged 8 years and older who were eligible for the DXA examination, 6350 had valid DXA-derived measures. In the subsample of 4756 participants 6 years and older who were eligible for the grip test in the 2011–2014 NHANES, 4355 participants completed the grip test. Of the 1220 Asian participants who were eligible for the acculturation questionnaire, 992 responded to the question about the language spoken at home. In a comparison of the characteristics between Asian participants who were excluded (*n* = 228) and those who were included (*n* = 992), the distributions of age, sex, family income, and use of an interpreter for the interview were similar (chi-square *p*-values > 0.8).

Among the five racial/ethnic groups examined, the Asian group had the lowest obesity prevalence (9.3%; 95% CI = 7.6, 11.0; Table 2), the lowest central obesity prevalence (18.8%; 96% CI = 15.4, 22.1), and the lowest elevated FMI prevalence (21.5%; 95% CI = 17.6%, 25.3%). However, the sex-specific analysis showed that the obesity, central obesity, and elevated FMI prevalences were not significantly lower in Asian American boys compared to non-Hispanic White boys. Rather, the central obesity and elevated FMI prevalence tended to be higher among Asian American boys aged 6–11 years compared to their non-Hispanic White counterparts. The low LBMI prevalence was the highest in the Asian group (42.2%; 95% CI = 37.6, 46.9), which indicates their low lean body mass.

Among the Asian American participants, 44.8% responded that they speak only English at home, 29.1% responded that they speak more English or English/non-English equally, and 26.1% responded that they speak more non-English or non-English only. As shown in Table 3, the relationship of acculturation with obesity was positive among boys (OR = 3.28; 95% CI = 1.63, 6.60) and with central obesity (OR = 3.04; 95% CI = 1.34, 6.90). Seventy-five percent of Asian American youth reported being born in the US. Asian American boys who were born in the US tended to be more likely to be obese than those born outside the US (OR = 1.70; 95% CI = 0.81, 3.57; see Supplementary materials Tables S1–S3).

Examining the two components of body composition, namely, elevated FMI prevalence and low LBMI prevalence, showed that they were not different by acculturation level, as indicated by either the home language indicator (Table 4) or birthplace (see Supplementary materials Tables S1–S3).

As presented in Table 5, Asian American youth reported a significantly lower frequency of eating frozen pizzas or meals compared to non-Hispanic White youth. In the five racial/ethnic groups, non-Hispanic Black youth presented the highest frequency of eating frozen pizzas or meals. The proportion of daily physical activity and muscle strength levels both tended to be lower in the Asian group than any other racial/ethnic group examined. 

As shown in Table 6, the more acculturated Asian American youth more frequently consumed frozen pizza or meals (1.4 more frozen meals/month; 95% CI = 1.2, 1.6) compared to their less acculturated counterparts. Acculturation was not associated with daily PA or grip strength.

## 4. Discussion

Using nationally representative data, this study found that Asian American girls, but not boys, were less likely to be obese compared to their non-Hispanic White American counterparts. Asian Americans also less frequently consumed convenience food. However, the more acculturated Asian American youth more frequently consumed convenience food. Furthermore, the more acculturated Asian American boys, but not girls, were more likely to be obese. Asian American youth were also less likely to engage in daily physical activity and had lower lean mass and lower muscle strength. Acculturation was not associated with daily physical activity, lean body mass, or muscle strength.

This is one of the first studies using a sufficiently large Asian youth oversample to allow for comparisons across US minority groups. Compared to other national studies, such as the Youth Risk Behavior Survey, the NHANES collects physical measures and asks unique survey questions, providing new, validated analyses that give strong evidence for a better understanding of obesity and obesogenic behaviors. This study found some better health measures (low obesity prevalence and less frequent convenience food consumption) and some worse health measures (lower muscle mass and lower daily physical activity prevalence) among Asian American youth compared to their non-Hispanic White counterparts. However, an in-depth analysis revealed that the obesity prevalence among Asian American boys was not much different from non-Hispanic White American boys. This finding, in conjunction with the changes in diet and low physicality, is particularly alarming because among Asian boys who are known to have lower lean body mass than White boys [32,33,34], the increased body weight implies a worse body composition and increased risks of hypertension and diabetes [12,13].

The application of the universal obesity definition across the racial/ethnic groups is advantageous in that an identical metric was used for the comparison of obesity prevalence. However, Deurenberge et al. [33] argued that a universal BMI threshold for the obesity definition across the ethnic groups, such as Asians, is inappropriate because BMI tends to underestimate body fat in Asians [32]. Recognizing this problem, a prior study [35] utilized a lower BMI threshold for Asian American adults (BMI ≥ 25 kg/m^2^) and revealed a higher obesity prevalence among Asian American adults than non-Hispanic White American adults. To evaluate excess body fat more appropriately among Asian youth, the utilization of an Asian-specific obesity definition should be considered. Hudda et al. [32] quantified the BMI adjustment needed for South Asian children in a United Kingdom child population. However, no Asian-specific reference curves or thresholds to define obesity have yet been established for youth in the US. This could partly be due to the lack of data regarding body composition in Asian American youth to support the need for an Asian-specific childhood obesity definition. Further research should follow to explore an appropriate Asian-specific childhood obesity definition in the US population. Nonetheless, these results call for closer attention to the obesity problem among Asian American boys.

It has been widely accepted that acculturation negatively affects the health of immigrants (“negative acculturation theory”) [36]. However, it has also been recognized that the health impact of acculturation is complex, varying by health outcomes, ethnicity, and gender, for example [37]. The current study suggests that as they become more acculturated, Asian American youth change their positive health behavior (i.e., less frequent convenience food consumption) in a negative direction. On the other hand, greater acculturation did not change their poorer health behavior (lower daily physical activity prevalence) in a positive direction. These findings generally support the “negative acculturation theory.” However, acculturation negatively influenced excess body weight only among Asian American boys, but not girls, which is in line with some prior study findings [38,39], but not all [40]. The sex difference in the acculturation effect could be due to the sex difference in the US cultural norms for body image: norms of muscularity for males and slimness for females [38]. A prior study [41] reported that among Asian US college students, males rated their current body size as smaller than ideal, while females rated their current body size as larger than ideal, presumably reflecting their desires for a large body size in males and a small body size in females. Parents also play a very influential role in the food preferences of their children [42]. Parents who are immigrants may be slower to recognize the risks for obesity in their sons than their daughters due to beliefs regarding body sizes around gender and parenting practices that are more indulgent and permissive with food consumption among boys [43]. Therefore, this study’s finding highlights that acculturation can affect health behaviors in different ways based on gender.

This study revealed that daily physical activity was lower among Asian Americans compared to non-Hispanic White Americans. The lack of integration of the Asian community could partly explain the lower participation in physical activity, such as sport [44]. Such lack of social integration could lead to the lack of parents’ awareness of the health risks associated with obesogenic environments in Western countries and the lack of information about the benefits of participating in sports or other extracurricular activities. Despite the possible effect of acculturation on physical activity, this study showed that acculturation was not associated with higher physical activity.

To better understand the association between acculturation and obesity-related behaviors, it is worth comparing Asian and Hispanic American youth. The two populations are similar in that both come from largely immigrant families, although the differences in anthropometric traits, shape, and timing of maturation should also be considered [45]. In this study sample, 21.1% of Hispanic American youth responded that they spoke only Spanish or more Spanish than English at home, while 26.1% of Asian American youth responded that they spoke non-English (presumably, an Asian language) only or more non-English than English in the home. Interestingly, these two ethnic groups presented a similar level of convenience food consumption. Although the data are not presented in the results above, the frequency of meals not prepared at home was also similar between the two groups (1.7 meals/week in the Asian group, 2.1 meals/week in the Hispanic group, 3.3 meals/week in the non-Hispanic White group, and 4.1 meals/week in the non-Hispanic Black group). Daily physical activity was slightly higher in the Hispanic group than in the Asian group. However, the obesity prevalence and the elevated FMI prevalence were approximately twice as high in the Hispanic group compared to the Asian group. This indirectly implies that the meals prepared at home in Asian American families could be healthier in terms of obesity prevention [7] compared to those prepared in Hispanic American families. A better understanding of dietary behaviors, food consumption, and diet quality among Asian American families could help to develop childhood obesity prevention strategies. This is especially important for families who are quickly acculturating.

Several limitations of this study should be acknowledged. First, although food consumption and physical activity behaviors perhaps differed by Asian ethnic subgroups [46], this study could not examine the differences between the subgroups due to the small sample size for each subgroup [25]. Second, self-reported diet and physical activity data are prone to measurement error [47,48]. In particular, the measurement error could differ by racial/ethnic groups, which may have caused differential misclassification errors. Third, the aerobic Physical Activity Guideline for youth aged 6 through 17 years (≥60 minutes of daily moderate to vigorous physical activity) was applied to children aged 2 to 5 years because NHANES data did not allow us to evaluate whether participants were active for ≥3 hours/day, which is a recommended level of physical activity for preschool-aged children [21]. Fourth, the spoken language at home alone may not equally represent acculturation levels achieved across Asian ethnic subgroups, particularly since English fluency differs by ethnic subgroups: e.g., 49% of Vietnamese and 45% of Chinese were not fluent in English, while only 19% of Asian Indians and 21% of Filipinos were not fluent in English [1]. However, when the birthplace (US vs. non-US) was used as an acculturation indicator, we found similar results with the language spoken at home. Years living in the US could not be used as an acculturation indicator because three in four participants were born in the US, and therefore, the number of years living in the US was strongly correlated with age. Lastly, this cross-sectional study does not provide evidence for a temporal relationship.

## 5. Conclusions

In conclusion, this study highlights the obesity problem among Asian American boys, which worsens with acculturation to America. The study results also suggest that Asian American youth consume less convenience food as compared to non-Hispanic White American youth, but acculturation to America may negatively influence their food choices.

## Figures and Tables

**Table 1:** Sample size from 2011–2018 US National Health and Nutrition Examination Survey (NHANES).

Age, yrs	Sex	Asian	White	Hispanic	Black	Other
				Unweighted *n*		
2–17	Both	1301	3397	3901	3247	917
2–5	Male	178	484	528	492	147
	Female	174	493	534	440	140
6–11	Male	243	716	808	654	183
	Female	226	651	812	656	197
12–17	Male	237	554	583	530	130
	Female	243	499	636	475	120

**Table 2:** Obesity, central obesity, elevated FMI, and low LBMI by race/ethnicity.

Age, yrs	Sex	Asian	White	Hispanic	Black	Other
		BMI ≥ 95th Percentile, weighted % (95% CI)				
2–17	Both	9.3 (7.6, 11.0)	14.5 (13.0, 16.0)	23.8 (22.1, 25.6)	21.1 (18.9, 23.3)	20.5 (16.1, 24.9)
2–5	Both	5.5 (2.7, 8.3)	8.6 (6.6, 10.5)	17.4 (14.8, 20.1)	11.3 (8.5, 14.1)	10.9 (5.5, 16.3)
	Male	6.6 (2.4, 10.8)	8.9 (6.2, 11.7)	19.3 (15.3, 23.4)	10.4 (6.6, 14.3)	10.1 (3.4, 16.9)
	Female	4.3 (0.4, 8.2)	8.2 (5.6, 10.8)	15.5 (11.7, 19.3)	12.1 (9.0, 15.2)	11.5 (2.9, 20.2)
6–11	Both	10.0 (6.7, 13.3)	14.7 (12.1, 17.3)	25.8 (23.5, 28.2)	21.9 (19.0, 24.8)	19.4 (13.7, 25.1)
	Male	14.4 (8.9, 19.9)	15.6 (12.6, 18.7)	26.7 (22.6, 30.8)	19.9 (16.6, 23.3)	23.8 (15.7, 31.9)
	Female	5.3 (2.2, 8.3)	13.8 (10.3, 17.3)	24.9 (21.5, 28.4)	23.9 (20.1, 27.7)	15.8 (9.4, 22.1)
12–17	Both	11.0 (8.1, 13.8)	17.6 (14.7, 20.6)	26.1 (22.9, 29.3)	26.3 (22.3, 30.3)	28.6 (20.1, 37.0)
	Male	14.5 (9.9, 19.2)	17.5 (13.9, 21.1)	26.9 (22.9, 31.0)	22.3 (18.1, 26.5)	31.4 (19.6, 43.2)
	Female	7.7 (4.4, 10.9)	17.8 (13.4, 22.1)	25.2 (21.0, 29.3)	30.7 (24.7, 36.7)	25.5 (14.7, 36.3)
**Waist Circumference ≥ 95th Percentile, weighted % (95% CI)**						
6–17	Both	18.8 (15.4, 22.1)	22.9 (20.1, 25.7)	31.7 (29.5, 33.8)	25.1 (22.7, 27.4)	27.6 (21.9, 33.2)
6–11	Both	16.5 (12.6, 20.3)	18.8 (15.6, 22.1)	29.3 (26.7, 31.9)	20.7 (18.4, 23.0)	23.3 (16.6, 30.1)
	Male	18.7 (13.0, 24.5)	15.7 (12.1, 19.3)	26.6 (22.4, 30.9)	16.4 (13.9, 18.9)	24.0 (15.4, 32.5)
	Female	14.0 (9.4, 18.6)	22.2 (17.7, 26.7)	32.1 (28.7, 35.5)	25.5 (21.1, 28.8)	22.8 (14.3, 31.3)
12–17	Both	21.0 (16.5, 25.5)	26.8 (23.0, 30.6)	34.2 (31.2, 37.3)	29.3 (25.9, 32.7)	32.6 (23.4, 41.7)
	Male	24.2 (18.2, 30.1)	24.3 (20.1, 28.4)	31.3 (26.9, 35.6)	21.3 (17.1, 25.5)	34.7 (24.0, 45.3)
	Female	18.0 (12.5, 23.5)	29.3 (24.1, 34.5)	37.4 (33.7, 41.0)	37.9 (33.1, 42.7)	30.4 (18.1, 42.6)
		**FMI ≥ 75th Percentile, weighted % (95% CI)**				
8–17	Both	21.5 (17.6, 25.3)	27.7 (24.7, 30.7)	40.1 (37.5, 42.7)	30.2 (27.1, 32.3)	34.6 (28.2, 41.0)
8–11	Both	24.8 (19.2, 30.3)	26.9 (22.4, 31.4)	42.2 (38.8, 45.6)	29.0 (25.2, 32.9)	30.4 (21.8, 38.9)
	Male	36.1 (27.5, 44.6)	28.3 (22.4, 34.3)	46.5 (41.5, 51.5)	28.1 (23.1, 33.2)	30.0 (19.2, 40.9)
	Female	12.3 (5.8, 18.7)	25.6 (20.0, 31.1)	37.5 (32.8, 42.3)	30.0 (24.0, 36.0)	30.7 (18.3, 43.2)
12–17	Both	19.4 (14.9, 24.0)	28.2 (24.2, 32.2)	38.5 (35.2, 41.9)	30.9 (26.8, 35.1)	37.5 (29.7, 45.4)
	Male	24.6 (17.9, 31.3)	29.1 (24.1, 34.1)	37.1 (32.7, 41.6)	26.4 (21.6, 31.2)	39.5 (28.7, 50.3)
	Female	15.6 (8.5, 20.6)	27.3 (22.1, 32.5)	40.1 (35.6, 44.6)	36.6 (29.4, 43.8)	35.3 (23.7, 47.0)
		**LBMI ≤ 25th Percentile, weighted % (95% CI)**				
8–17	Both	42.2 (37.6, 46.9)	27.1 (24.2, 29.9)	23.8 (21.3, 26.3)	15.1 (12.9, 17.3)	24.2 (17.3, 31.0)
8–11	Both	45.2 (37.5, 53.0)	31.3 (27.3, 35.2)	27.4 (23.8, 30.9)	17.2 (14.0, 20.4)	33.3 (24.8, 41.8)
	Male	47.6 (37.5, 57.6)	32.9 (27.1, 38.6)	27.7 (22.2, 33.1)	18.7 (14.0, 23.4)	36.2 (24.0, 48.4)
	Female	42.7 (33.9, 51.4)	29.7 (23.7, 35.7)	27.0 (22.7, 31.3)	15.7 (10.8, 20.6)	30.5 (18.0, 43.1)
12–17	Both	40.4 (34.3, 46.5)	24.4 (20.7, 28.1)	21.2 (18.2, 24.3)	13.8 (10.9, 16.6)	17.6 (9.2, 26.0)
	Male	40.3 (31.4, 49.2)	23.9 (19.6, 28.1)	19.6 (16.1, 23.1)	15.5 (11.8, 19.2)	17.5 (4.7, 30.3)
	Female	40.5 (32.5, 48.5)	24.9 (19.6, 30.2)	23.1 (18.4, 27.8)	11.9 (8.3, 15.4)	17.7 (5.0, 30.4)

BMI, body mass index; CI, confidence interval; FMI, fat mass index; LBMI, lean body mass index.

**Table 3:** Associations of acculturation with obesity and central obesity in Asian American children.

Predictor	BMI ≥ 95th Percentile in Males (*n* = 476)	BMI ≥ 95th Percentile in Females (*n* = 442)	Waist Circumference ≥ 95th Percentile in Males (*n* = 385)	Waist Circumference ≥ 95th Percentile in Females (*n* = 368)
		OR (95% CI)		
Age: 6–11 vs. 3–5 yrs	1.97 (0.77, 5.07)	1.71 (0.32, 9.06)	NA	NA
Age: 12–17 vs. 3–5 yrs (vs. 6–11 yrs for waist circumference)	1.77 (0.69, 4.49)	2.24 (0.55, 9.02)	1.27 (0.73, 2.21)	1.43 (0.80, 2.58)
Family income: Middle vs. low	1.11 (0.62, 2.00)	1.20 (0.44, 3.28)	1.35 (0.70, 2.59)	0.53 (0.23, 1.21)
Family income: High vs. low	0.68 (0.37, 1.26)	0.65 (0.26, 1.61)	1.08 (0.62, 1.88)	0.47 (0.23, 0.99)
Home language: more English or English/non-English equally vs. more non-English or non-English only	2.61 (1.36, 4.97)	0.83 (0.39, 1.78)	2.50 (1.16, 5.36)	1.83 (0.77, 4.33)
Home language: English only vs. more non-English or non-English only	3.28 (1.63, 6.60)	0.78 (0.32, 1.93)	3.04 (1.34, 6.90)	1.52 (0.62, 3.74)

BMI, body mass index; CI, confidence interval; NA, not applicable because the multivariable logistic regression model did not include the parameter; OR, odds ratio.

**Table 4:** Associations of acculturation with elevated FMI and low LBMI in Asian American children.

Predictor	FMI ≥ 75th Percentile in Males (*n* = 272)	FMI ≥ 75th Percentile in Females (*n* = 255)	LBMI ≤ 25th Percentile (*n* = 529)
		OR (95% CI)	
Sex: male vs. female	NA	NA	1.07 (0.77, 1.50)
Age: 12–17 vs. 8–11 yrs	0.55 (0.33, 0.90)	1.32 (0.59, 2.97)	0.86 (0.55, 1.35)
Family income: middle vs. low	1.70 (0.76, 3.84)	1.31 (0.42, 4.07)	1.45 (0.92, 2.28)
Family income: high vs. low	2.05 (1.01, 4.16)	0.59 (0.18, 1.89)	1.30 (0.74, 2.31)
Home language: more English or English/non-English equally vs. more non-English or non-English only	1.45 (0.80, 2.62)	1.12 (0.50, 2.91)	1.12 (0.68, 1.86)
Home language: English only vs. more non-English or non-English only	1.21 (0.69, 2.14)	0.62 (0.27, 1.45)	1.35 (0.85, 2.13)

CI, confidence interval; FMI, fat mass index; LBMI, lean body mass index; NA, not applicable because the multivariable logistic regression model did not include the parameter; OR, odds ratio.

**Table 5:** Convenience food consumption, daily physical activity, and muscle strength by race/ethnicity.

Age, yrs	Sex	Asian	White	Hispanic	Black	Other
		Number of Frozen Pizzas or Meals Eaten per Month, weighted mean (95% CI)				
2–17	Both	1.7 (1.4, 2.0)	3.3 (3.0, 3.5)	2.1 (1.8, 2.4)	4.1 (3.5, 4.6)	3.4 (2.9, 3.8)
2–5	Both	0.8 (0.6, 1.1)	2.7 (2.3, 3.0)	2.0 (1.4, 2.5)	4.2 (3.3, 5.1)	2.7 (2.1, 3.3)
	Male	0.7 (0.4, 0.9)	3.1 (2.5, 3.6)	1.8 (1.1, 2.6)	4.3 (3.3, 5.2)	3.4 (2.4, 4.3)
	Female	1.0 (0.6, 1.4)	2.3 (1.9, 2.7)	2.1 (1.5, 2.7)	4.1 (2.8, 5.4)	2.0 (1.5, 2.6)
6–11	Both	2.0 (1.5, 2.6)	3.1 (2.7, 3.5)	2.1 (1.7, 2.5)	3.6 (3.0, 4.1)	3.3 (2.3, 4.2)
	Male	2.2 (1.3, 3.1)	3.4 (2.8, 4.0)	2.4 (1.7, 3.1)	3.7 (3.0, 4.4)	4.1 (2.3, 6.0)
	Female	1.8 (1.0, 2.6)	2.7 (2.2, 3.1)	1.7 (1.3, 2.0)	3.4 (2.8, 4.1)	2.5 (1.5, 3.5)
12–17	Both	2.0 (1.5, 2.4)	3.8 (3.4, 2.3)	2.3 (1.9, 2.7)	4.5 (3.7, 5.3)	4.0 (2.9, 5.0)
	Male	2.3 (1.6, 3.0)	4.2 (3.6, 4.9)	2.8 (2.0, 3.5)	5.3 (4.1, 6.5)	4.6 (2.9, 6.2)
	Female	1.6 (1.1, 2.1)	3.4 (2.7, 4.0)	1.8 (1.4, 2.2)	3.7 (2.7, 4.6)	3.4 (2.4, 4.3)
		**Daily PA, weighted % (95% CI)**				
2–17	Both	40.0 (35.8, 44.2)	43.7 (41.5, 45.9)	43.1 (40.3, 45.8)	46.2 (43.7, 48.8)	43.2 (39.0, 47.4)
2–5	Both	73.0 (66.1, 79.9)	76.6 (72.7, 80.5)	71.9 (68.0, 75.9)	76.8 (72.8, 80.8)	73.5 (64.9, 82.0)
	Male	75.8 (67.5, 84.1)	77.6 (73.1, 82.0)	72.8 (67.5, 78.2)	80.2 (75.7, 84.6)	76.2 (65.3, 87.0)
	Female	69.8 (60.4, 79.2)	75.6 (70.5, 80.7)	71.0 (66.6, 75.4)	73.4 (67.6, 79.3)	70.7 (60.9, 80.6)
6–11	Both	55.3 (49.2, 61.5)	59.4 (55.5, 63.2)	57.4 (53.2, 61.6)	64.3 (60.4, 68.1)	51.9 (44.4, 59.5)
	Male	59.8 (51.8, 67.9)	64.3 (59.8, 68.8)	60.7 (55.6, 65.9)	65.6 (60.5, 70.7)	53.3 (43.6, 63.1)
	Female	50.4 (42.1, 58.7)	54.1 (49.1, 59.0)	53.9 (49.0, 58.9)	62.9 (58.5, 67.3)	50.8 (41.5, 60.0)
12–17	Both	5.4 (3.3, 7.4)	10.1 (8.1, 12.2)	7.0 (5.3, 8.7)	10.7 (8.2, 13.2)	11.1 (5.8, 16.3)
	Male	6.4 (3.5, 9.4)	13.5 (10.2, 16.9)	9.4 (6.3, 12.6)	13.0 (9.6, 16.4)	14.1 (6.2, 22.0)
	Female	4.3 (1.5, 7.1)	6.8 (4.2, 9.4)	4.4 (2.8, 6.0)	8.2 (5.4, 11.0)	7.7 (1.8, 13.5)
		**Grip Strength in kg, weighted mean (95% CI)**				
6–17	Both	42.7 (38.6, 46.8)	45.9 (42.7, 49.1)	44.0 (41.4, 46.6)	49.8 (45.9, 53.6)	45.0 (34.8, 55.2)
6–11	Both	26.6 (24.6, 28.5)	30.0 (28.0, 31.9)	29.1 (27.6, 30.6)	32.9 (31.5, 34.4)	29.4 (25.3, 33.6)
	Male	27.0 (24.2, 29.8)	30.2 (28.1, 32.3)	29.5 (27.7, 31.3)	33.3 (31.0, 35.6)	30.8 (24.7, 36.9)
	Female	26.1 (23.1, 29.1)	29.7 (26.4, 33.1)	28.7 (26.2, 31.2)	32.5 (30.5, 34.6)	28.3 (23.3, 33.4)
12–17	Both	57.0 (52.4, 64.6)	60.8 (57.5, 64.1)	59.5 (55.1, 64.0)	65.7 (62.0, 69.4)	63.3 (54.3, 72.3)
	Male	64.6 (57.3, 71.9)	68.8 (62.6, 75.0)	67.9 (62.7, 73.2)	72.7 (67.2, 78.2)	71.8 (63.2, 80.5)
	Female	49.2 (44.9, 53.5)	53.6 (49.9, 57.3)	50.1 (46.9, 53.2)	57.7 (54.5, 60.9)	54.1 (43.9, 64.3)

PA, physical activity.

**Table 6:** Associations of acculturation with convenience food consumption, daily physical activity, and muscle strength in Asian American children.

Predictor	Frozen Pizzas or Meals Eaten, Meals/Month (*n* = 946)	Daily PA (*n* = 992)	Grip Strength, kg (*n* = 334)
	Estimate (95% CI)	OR (95% CI)	Estimate (95% CI)
Sex: male vs. female	0.3 (0.1, 0.5)	1.45 (1.09, 1.93)	10.5 (9.2, 11.9)
Age: 6–11 vs. 3–5 yrs	1.0 (1.0, 1.0)	0.43 (0.30, 0.61)	NA
Age: 12–17 vs. 3–5 yrs (vs. 6–11 yrs for grip strength)	0.8 (0.7, 1.0)	0.02 (0.01, 0.04)	30.4 (29.1, 31.7)
Family income: middle vs. low	0.2 (0.1, 0.4)	1.23 (0.75, 2.02)	−2.1 (−3.6, −0.6)
Family income: high vs. low	−0.4 (−0.5, −0.2)	1.38 (0.88, 2.17)	−0.5 (−2.5, 1.4)
Home language: more English or English/non-English equally vs. more non-English or non-English only	1.1 (0.9, 1.2)	1.31 (0.81, 2.10)	1.0 (−1.0, 3.0)
Home language: English only vs. more non-English or non-English only	1.4 (1.2, 1.6)	1.07 (0.68, 1.71)	1.1 (−0.2, 2.5)
Intercept	0.1 (−0.1, 0.2)	NA	21.4 (19.3, 23.5)

CI, confidence interval; NA, not applicable; OR, odds ratio; PA, physical activity.

## Supplementary Materials

The following are available online at https://www.mdpi.com/1660-4601/17/17/6187/s1, Table S1: Associations of birthplace with obesity and central obesity in Asian American children, Table S2: Associations of birthplace with elevated FMI and low LBMI in Asian American children, Table S3: Associations of birthplace with convenience food consumption, daily physical activity, and muscle strength in Asian American children.
