# Supplementary material for: Body Composition, Physical Activity, and Convenience Food Consumption among Asian American Youth: 2011–2018 NHANES
Source: Int J Environ Res Public Health. 2020 Aug 26;17(17):6187. doi: 10.3390/ijerph17176187 (PMC7504455; doi:10.3390/ijerph17176187)
Supplement: Supplementary file 1 [file ijerph-17-06187-s001.pdf]

Supplementary Data

Table S1. Associations of birthplace with obesity and central obesity in Asian American children.

	BMI ≥ 95th percentile *in males* (n = 608)	BMI ≥ 95th percentile *in females* (n = 586)	Waist circumference ≥ 95th percentile *in males* (n = 454)	Waist circumference ≥ 75th percentile *in females* (n = 433)
	OR (95% CI)	OR (95% CI)	OR (95% CI)	OR (95% CI)
Age: 6–11 vs. 3-5 yrs	2.58 (1.13, 5.86)	1.11 (0.34, 3.65)	NA	NA
Age: 12–17 vs. 3-5 yrs (vs. 6-11 yrs for waist circumference)	2.51 (1.06, 5.97)	1.60 (0.59, 4.34)	1.42 (0.86, 2.35)	1.36 (0.78, 2.38)
Family income: Middle vs. low	0.95 (0.49, 1.83)	1.80 (0.54, 5.99)	1.21 (0.67, 2.21)	0.61 (0.28, 1.31)
Family income: High vs. low	0.62 (0.32, 1.18)	0.62 (0.22, 1.71)	1.10 (0.61, 1.97)	0.52 (0.26, 1.34)
Birthplace: U.S. vs. non-U.S.	1.70 (0.81, 3.57)	0.97 (0.40, 2.38)	1.27 (0.66, 2.45)	1.06 (0.54, 2.08)

BMI, body mass index; CI, confidence interval; FMI, fat mass index; LBMI, lean body mass index; NA, not applicable because the multivariable logistic regression model did not include the parameter; OR, odds ratio.

Table S2. Associations of birthplace with elevated FMI and low LBMI in Asian American children.

	FMI ≥ 75th percentile *in males* (n = 305)	FMI ≥ 75th percentile *in females* (n = 285)	LBMI ≤ 25th percentile (n = 595)
	OR (95% CI)	OR (95% CI)	OR (95% CI)
Sex: male vs. female	NA	NA	1.08 (0.75, 1.54)
Age: 12–17 vs. 8–11 yrs	0.60 (0.37, 0.99)	1.16 (0.51, 2.66)	0.84 (0.56, 1.27)
Family income: Middle vs. low	1.63 (0.75, 3.55)	1.42 (0.39, 5.16)	1.30 (0.80, 2.09)
Family income: High vs. low	1.64 (0.83, 3.23)	0.59 (0.16, 2.16)	1.33 (0.76, 2.34)
Birthplace: U.S. vs. non-U.S.	1.09 (0.53, 2.24)	0.91 (0.35, 2.37)	1.17 (0.78, 1.76)

CI, confidence interval; FMI, fat mass index; LBMI, lean body mass index; NA, not applicable because the multivariable logistic regression model did not include the parameter; OR, odds ratio.

Table S3. Associations of birthplace with convenience food consumption, daily physical activity, and muscle strength in Asian American children

	**Frozen pizzas or meals eaten, meals/month (n = 1252)**	**Daily PA (n = 1301)**	**Grip strength, kg (n = 456)**
	Estimate (95% CI)	OR (95% CI)	Estimate (95% CI)
Sex: male vs. female	0.3 (-0.2, 0.8)	1.45 (1.10, 1.91)	8.6 (6.4, 10.7)
Age: 6-11 vs. 3-5 yrs	1.2 (0.5, 1.8)	0.46 (0.34, 0.63)	NA
Age: 12-17 vs. 3-5 yrs (vs. 6-11 yrs for grip strength)	1.0 (0.5, 1.6)	0.02 (0.01, 0.04)	30.6 (28.4, 32.8)
Family income: Middle vs. low	0.1 (-0.9, 1.1)	1.02 (0.62, 1.69)	-1.5 (-4.2, -1.3)
Family income: High vs. low	-0.1 (-1.0, 0.8)	0.95 (0.64, 1.41)	-0.7 (-3.9, 2.5)
Birthplace: U.S. vs. non-U.S.	-0.4 (-1.1, 0.3)	1.28 (0.85, 1.93)	-0.2 (-2.9, 2.6)
Intercept	1.0 (0.1, 1.9)	NA	23.0 (18.8, 27.2)

CI, confidence interval; NA, not applicable; OR, odds ratio; PA, physical activity.
